# *Sensors* Best Paper Award 2013

**DOI:** 10.3390/s130202113

**Published:** 2013-02-05

**Authors:** Vittorio M.N. Passaro, W. Rudolf Seitz, Assefa M. Melesse, Alexander Star, Ophelia Han

**Affiliations:** 1 Dipartimento di Ingegneria Elettrica e dell'Informazione, Politecnico di Bari, Via Edoardo Orabona n. 4, 70125 Bari, Italy; E-Mail: passaro@deemail.poliba.it; Tel.: +39-08059-63850; Fax: +39-08059-63410; 2 Analytical Chemistry, Department of Chemistry, University of New Hampshire, Durham, NH 03824, USA; E-Mail: wrs@cisunix.unh.edu; Tel.: +1-603-862-2408; Fax: +1-603-862-4278; 3 Department of Earth and Environment, ECS 339, Florida International University, 11200 SW 8th Street, Miami, FL 33199, USA; E-Mail: melessea@fiu.edu; Tel.: +1-305-348-6518; Fax: +1-305-348-6137; 4 Department of Chemistry, University of Pittsburgh, 219 Parkman Avenue, Pittsburgh, PA 15260, USA; E-Mail: astar@pitt.edu; Tel.: +1-412-624-6493; Fax: +1-412-624-4027; 5 MDPI AG, Postfach, CH-4005 Basel, Switzerland and MDPI Branch Office, Beijing 101101, China; E-Mail: ophelia.han@mdpi.com; Tel./Fax: +86-10-8152-1170

Since 2011, *Sensors* has instituted an annual award to recognize outstanding papers that are related to sensing technologies and applications and meet the aims, scope and high standards of this journal [1,2]. This year, nominations were made by the Section Editor-in-Chiefs of *Sensors* from among all the papers published in 2009. Reviews and full research articles were considered separately. We are pleased to announce that the following eight papers have won the *Sensors* Best Paper Award in 2013:

## Article Award

### 1^st^ Prize

**Sabine Achmann, Gunter Hagen, Jaroslaw Kita, Itamar M. Malkowsky, Christoph Kiener and Ralf Moos**

Metal-Organic Frameworks for Sensing Applications in the Gas Phase

*Sensors* **2009**, *9*(3), 1574-1589; doi:10.3390/s90301574

Available online: http://www.mdpi.com/1424-8220/9/3/1574

### 2^nd^ Prize

**Alimujiang Fulati, Syed M. Usman Ali, Muhammad Riaz, Gul Amin, Omer Nur and Magnus Willander**

Miniaturized pH Sensors Based on Zinc Oxide Nanotubes/Nanorods

*Sensors* **2009**, *9*(11), 8911-8923; doi:10.3390/s91108911

Available online: http://www.mdpi.com/1424-8220/9/11/8911

### 3^rd^ Prize

**Filiberto Chiabrando, Roberto Chiabrando, Dario Piatti and Fulvio Rinaudo**

Sensors for 3D Imaging: Metric Evaluation and Calibration of a CCD/CMOS Time-of-Flight Camera

*Sensors* **2009**, *9*(12), 10080-10096; doi:10.3390/s91210080

Available online: http://www.mdpi.com/1424-8220/9/12/10080

### 4^th^ Prize

**Litao Liu, Xiongying Ye, Kang Wu, Rui Han, Zhaoying Zhou and Tianhong Cui**

Humidity Sensitivity of Multi-Walled Carbon Nanotube Networks Deposited by Dielectrophoresis

*Sensors* **2009**, *9*(3), 1714-1721; doi:10.3390/s90301714

Available online: http://www.mdpi.com/1424-8220/9/3/1714

### 5^th^ Prize

**Andrea Lingua, Davide Marenchino and Francesco Nex**

Performance Analysis of the SIFT Operator for Automatic Feature Extraction and Matching in Photogrammetric Applications

*Sensors* **2009**, *9*(5), 3745-3766; doi:10.3390/s90503745

Available online: http://www.mdpi.com/1424-8220/9/5/3745

## Review Award

### 1^st^ Prize

**Alphus D. Wilson and Manuela Baietto**

Applications and Advances in Electronic-Nose Technologies

*Sensors* **2009**, *9*(7), 5099-5148; doi:10.3390/s90705099

Available online: http://www.mdpi.com/1424-8220/9/7/5099

### 2^nd^ Prize

**Chuji Wang and Peeyush Sahay**

Breath Analysis Using Laser Spectroscopic Techniques: Breath Biomarkers, Spectral Fingerprints, and Detection Limits

*Sensors* **2009**, *9*(10), 8230-8262; doi:10.3390/s91008230

Available online: http://www.mdpi.com/1424-8220/9/10/8230

### 3^rd^ Prize

**Tianyou Zhai, Xiaosheng Fang, Meiyong Liao, Xijin Xu, Haibo Zeng, Bando Yoshio and Dmitri Golberg**

A Comprehensive Review of One-Dimensional Metal-Oxide Nanostructure Photodetectors

*Sensors* **2009**, *9*(8), 6504-6529; doi:10.3390/s90806504

Available online: http://www.mdpi.com/1424-8220/9/8/6504

The prize awarding committee merits the article “Metal-Organic Frameworks for Sensing Applications in the Gas Phase” as “involving a new type of material as the adsorbent in gas sensors”. Both of the reviews “Applications and Advances in Electronic-Nose Technologies” and “Breath Analysis Using Laser Spectroscopic Techniques: Breath Biomarkers, Spectral Fingerprints, and Detection Limits” “…introduced and explained their subject very nicely, while giving thorough, useful coverage of the literature.”

These eight exceptional papers are valuable contributions to *Sensors* and the sensing field. On behalf of the Prize Awarding Committee and the Editorial Board of *Sensors*, we would like to congratulate these eight teams for their excellent work. In recognition for their accomplishment, Drs. Sabine Achmann, Alimujiang Fulati, Dario Piatti, Xiongying Ye and Davide Marenchino will receive 1000 CHF, 800 CHF, 600 CHF, 400 CHF and 200 CHF, respectively, and the privilege of publishing an additional open access format paper of their choice free of charge in *Sensors* in 2013. Drs. Alphus D. Wilson, Chuji Wang, Tianyou Zhai and Xiaosheng Fang will be awarded the privilege of publishing an additional research paper free of charge in open access format in *Sensors*.

Prize Awarding Committee

*Editor-in-Chief*, Section ‘Physical Sensors’

**Dr. Vittorio M.N. Passaro**

Dipartimento di Ingegneria Elettrica e dell'Informazione, Politecnico di Bari, Via Edoardo Orabona n. 4, 70125 Bari, Italy

Tel. +39-08059-63850; Fax: +39-08059-63410

Website: http://dee.poliba.it/photonicsgroup

E-Mail: passaro@deemail.poliba.it

*Editor-in-Chief*, Section ‘Chemical Sensors’

**Prof. Dr. W. Rudolf Seitz**

Analytical Chemistry, Department of Chemistry, University of New Hampshire, Durham, NH 03824, USA

Tel. +1-603-862-2408; Fax: +1-603-862-4278

Website: http://www.unh.edu/chemistry/faculty/seitz_w.html

E-Mail: wrs@cisunix.unh.edu

*Editor-in-Chief*, Section ‘Remote Sensors’

**Dr. Assefa M. Melesse**

Department of Environmental Studies, ECS 339, Florida International University, 11200 SW 8th Street, Miami, FL 33199, USA

Tel. +1-305-348-6518; Fax: +1-305-348-6137

Website: http://www.fiu.edu/∼melessea/

E-Mail: assefa.melesse@fiu.edu

*Editor-in-Chief*, Section ‘Biosensors’

**Dr. Alexander Star**

Department of Chemistry, University of Pittsburgh, 219 Parkman Avenue, Pittsburgh, PA 15260, USA

Tel. +1-412-624-6493; Fax: +1-412-624-4027

Website: http://www.pitt.edu/∼astar/

E-Mail: astar@pitt.edu

Managing Editor

**Dr. Ophelia Han**

MDPI Beijing Office, Suite 2011, Ruidu International Center, Cuijingbeili No 1, Tongzhou District, Beijing 101101, China

E-Mail: ophelia.han@mdpi.com
